# The effect of Zhangfei/CREBZF on cell growth, differentiation, apoptosis, migration, and the unfolded protein response in several canine osteosarcoma cell lines

**DOI:** 10.1186/s12917-015-0331-y

**Published:** 2015-02-07

**Authors:** Rui Zhang, Douglas H Thamm, Vikram Misra

**Affiliations:** Department of Microbiology, Western College of Veterinary Medicine, University of Saskatchewan, Saskatoon, SK Canada; Flint Animal Cancer Center, Colorado State University, Fort Collins, CO USA; Present address: Department of Basic Veterinary Medicine, College of Veterinary Medicine, China Agricultural University, Beijing, China

**Keywords:** Canine osteosarcoma, Zhangfei/CREBZF, p53, Apoptosis, Osteocalcin

## Abstract

**Background:**

We had previously shown that the bLZip domain-containing transcription factor, Zhangfei/CREBZF inhibits the growth and the unfolded protein response (UPR) in cells of the D–17 canine osteosarcoma (OS) line and that the effects of Zhangfei are mediated by it stabilizing the tumour suppressor protein p53. To determine if our observations with D-17 cells applied more universally to canine OS, we examined three other independently isolated canine OS cell lines—Abrams, McKinley and Gracie.

**Results:**

Like D–17, the three cell lines expressed p53 proteins that were capable of activating promoters with p53 response elements on their own, and synergistically with Zhangfei. Furthermore, as with D–17 cells, Zhangfei suppressed the growth and UPR-related transcripts in the OS cell lines. Zhangfei also induced the activation of osteocalcin expression, a marker of osteoblast differentiation and triggered programmed cell death.

**Conclusions:**

Osteosarcomas are common malignancies in large breeds of dogs. Although there has been dramatic progress in their treatment, these therapies often fail, leading to recurrence of the tumour and metastatic spread. Our results indicate that induction of the expression of Zhangfei in OS, where p53 is functional, may be an effective modality for the treatment of OS.

## Background

Osteosarcoma (OS) is the most common primary malignant bone tumour in children and adolescents, although its incidence in dogs is ten times greater than in humans [1]. Spontaneously occurring osteosarcomas in dogs are an ideal model for cancer research due to their anatomical and physiological similarities with human counterparts (reviewed by [2-4]).

We had previously shown that the basic leucine zipper (bLZip) domain-containing transcription factor, Zhangfei/CREBZF/SMILE inhibits the growth and the unfolded protein response (UPR) in the D–17 canine osteosarcoma (OS) cell line [5] and that the effects of Zhangfei are mediated by stabilizing the tumour suppressor protein p53 [6]. To determine if our observations with D–17 cells applied more universally to canine OS, we examined three other independently isolated canine OS cell lines—Abrams, McKinley [7-10] and Gracie [11]. The purpose of this study was to determine the inhibitory role of Zhangfei in these OS cell lines by exploring its potential involvement in growth, differentiation, apoptosis, and metastasis.

Zhangfei was initially identified through its interaction with the host cell factor (HCF1) a protein required for the initiation of herpes simplex virus gene expression [12]. Unlike other bLZip transcription factors, Zhangfei appears to be incapable of binding to consensus bLZip response elements as a homodimer [13]. Instead, it fulfills its role in transcriptional regulation by hetero-dimerizing with and modulating other transcription factors or signaling molecules, such as Luman/CREB3 [14], Xbp1 [15], ATF4 [16], SMAD 1,5,8 [17], herpes simplex virus VP16 [18], and p53 [19].

## Results and discussion

### All four canine OS cells lines express functional p53

**Figure 1:**
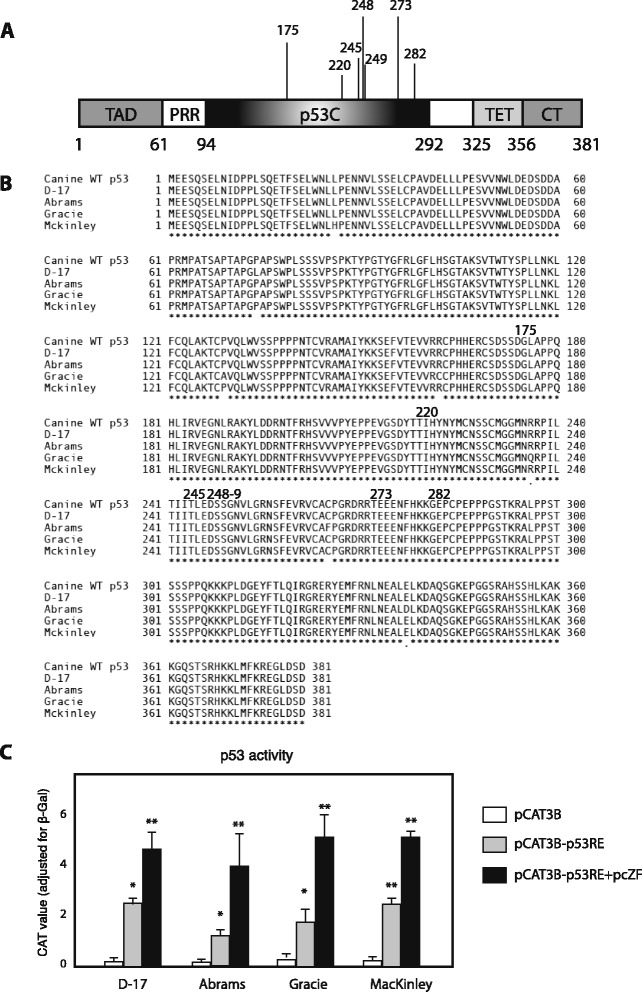
**p53 in dog osteosarcoma cell lines. (A)** Schematic structure of full-length p53. TAD: N-terminal transactivation domain; PRR: proline-rich region; p53C: central DNA-binding domain; TET: tetramerization domain; CT: extreme carboxyl terminus. p53C is the domain where most cancer-associated p53 mutations are located. The numbers below the diagram indicate amino acid residues delineating the domains and numbers above the diagram represent the residues with highest frequency of oncogenic missense mutations [20]. **(B)** Derived amino acid sequence alignment of p53s from 4 dog OS cell lines and dog wild-type p53. The residues that have high mutant frequency were marked above the diagram. Accession numbers: KP279761, KP279762, KP279763, KP279764 **(C)** p53 proteins of dog OS cell lines have transcriptional activity, and Zhangfei enhances p53-dependent transactivation. D–17, Abrams, McKinley, and Gracie cells were transfected with 0.5 μg of pCAT3B or pCAT3B-p53RE, in the presence or absence of 1 μg of pcZF. 24 h after transfection, the CAT activity was determined. Values represented the relative CAT activity (adjusted by β-galactosidase) of different treatments. Standard deviations from means of three individual experiments are shown. Significance of differences of the means (*P < 0.05, **P < 0.01) were determined using ANOVA.

To confirm the effects of Zhangfei we had observed in D–17 OS cells we examined three other canine OS cell lines. We have shown that Zhangfei exerts its effect on cell growth and the UPR by stabilizing p53 [6] and it therefore has no effect on cancer cells that do not possess functional p53. To assess the status of p53 in the canine cell lines we amplified p53 transcripts from the cells using PCR and determined the nucleotide sequences of the products. Figure 1B shows the derived amino acid sequences of p53 from the cell lines and the reference sequence from the canine genome database. All four cell lines contained transcripts for p53 that, with the exception of a few amino acid variations, were identical to the reference sequence. None of the amino acid polymorphisms in the sequences were at positions identified as important for p53 function [20] (Figure 1A,B).

To determine if the p53 proteins in the cell lines were functionally active, we transfected the cells with a plasmid that expressed the reporter protein chloramphenicol acetyl transferase (CAT) regulated by a promoter with two copies of a p53 response element (pCATp53RE). As a negative control, cells were transfected with a plasmid (pCAT3B) without the response elements. Parallel cultures were transfected with a plasmid expressing Zhangfei. Figure 1C shows that expression of CAT was activated in all four cell lines in a p53 response element–dependent manner and that the presence of Zhangfei enhanced expression.

### Cellular outcome following ectopic expression of Zhangfei: growth arrest, apoptosis and differentiation

**Figure 2:**
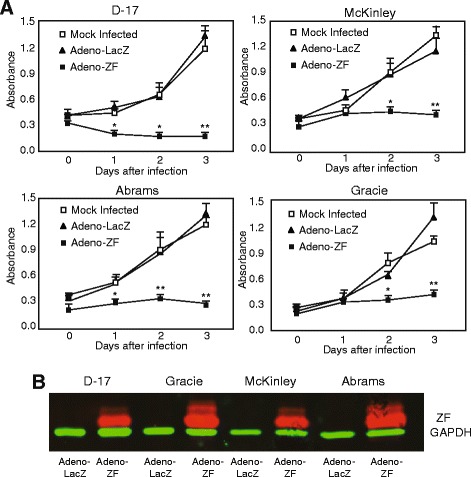
**Ectopic expression of Zhangfei suppresses cell growth in canine osteosarcomas.** D–17, Abrams, McKinley, and Gracie canine OS cells were mock-infected or infected with adenovirus vectors expressing either Zhangfei (Adeno-ZF) or β-galactosidase (Adeno-LacZ) and growth rates were measured **(A)** by absorbance at 405 nm with WST-1 at different time points after infection. Error bars indicate standard deviations from means of three individual experiments. Standard deviations from means of three individual experiments are shown and significance (*P < 0.05, **P < 0.01) was determined using ANOVA. **(B)**. Zhangfei was detected by immunoblotting using antiserum against Zhangfei.

**Figure 3:**
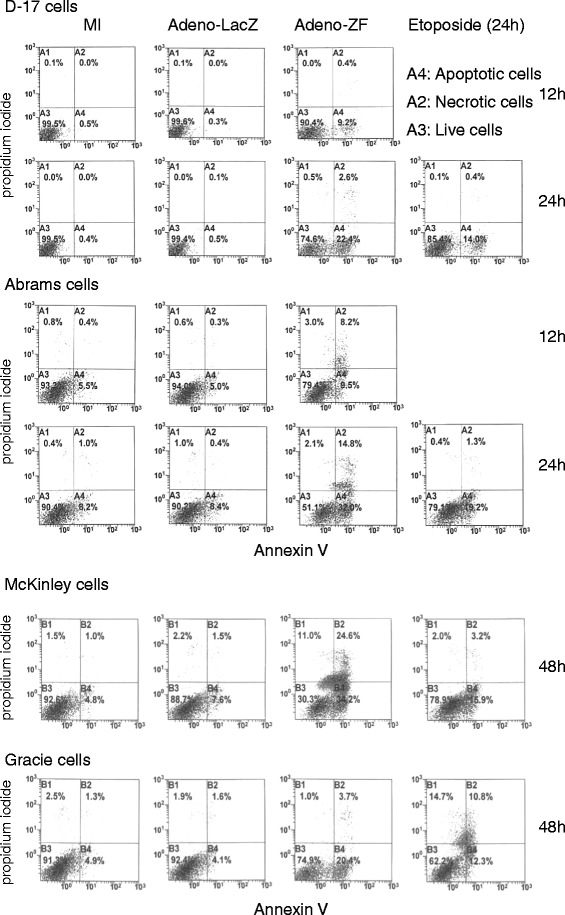
**Zhangfei causes canine osteosarcoma cells to commit apoptosis.** D–17, Abrams, McKinley and Gracie cells, mock-infected or infected with Adeno-ZF or Adeno-LacZ or treated with 50 μM etoposide (positive control) were stained with fluorescent Annexin V and propidium iodide. Unstained cells or cells staining with either or both dyes were enumerated by FACS. A4 represents the percentage of total cells undergoing apoptosis. For McKinley and Gracie only reading for 48 hr are shown. Differences between LacZ and ZF-expressing cells were more pronounced at that time-point.

We next compared the effect of Zhangfei on the growth characteristics of Abrams, McKinley, and Gracie cells with its effect on D–17 cells. The cells were infected with adenovirus expressing either Zhangfei (Adeno-ZF) or the control protein β-galactosidase (Adeno-LacZ). Cell growth was monitored by the WST-1 Cell Proliferation Assay. In agreement with previous results, all four Adeno-ZF-infected cells failed to divide as early as day 1 after infection as determined by their ability to convert WST-1 Cell Proliferation reagent and absorb light at 405 nm. Mock infected cells continued to grow for three days and the growth of Adeno-LacZ-infected cells was indistinguishable from mock-infected cells (Figure 2). Apoptosis was induced in all four cell lines as a result of Zhangfei expression (Figure 3). D–17 and Abrams appeared to be more sensitive as cultures had substantial numbers of apoptotic cells at 24 hr. The response in McKinley and Gracie, was slower with cultures showing 20 to 34% apoptotic cells by 48 hr. Since the level of p53 in response to Zhangfei was similar in all the cell lines the quantitative differences in the induction of apoptosis suggests that Zhangfei may have effects that are, at least partially, independent of p53.

**Figure 4:**
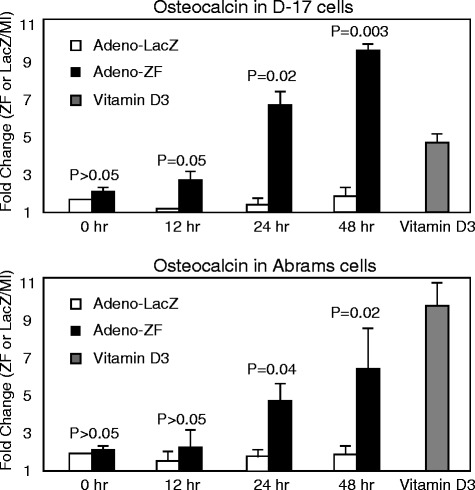
**Zhangfei induces differentiation of canine osteosarcoma cells.** D–17 and Abrams cells were either mock-infected or infected with Adeno-ZF or Adeno-LacZ. The positive control cells were treated with 10^-5^ mM vitamin D3. The mRNA levels of osteoblast differentiation marker (osteocalcin) were estimated by qRT-PCR. Standard deviations from means and P values as calculated using a Student *T*-test are shown.

Since the effect of Zhangfei was most dramatic on D–17 and Abrams cells we selected them for further analysis. Zhangfei may stop cell growth by inducing differentiation and/or causing apoptosis, we therefore performed a transcript level analysis of the OS differentiation marker**—**osteocalcin [21] in D–17 and Abrams cells infected with either Adeno-ZF or Adeno-LacZ. Compared with LacZ-expressing and even vitamin D3-treated cells (negative and positive controls, respectively), Zhangfei significantly increased the expression of osteocalcin transcripts in a time-dependent manner (Figure 4).

### Expression of Zhangfei inhibits the ability of canine osteosarcoma cells to close a scratch wound

**Figure 5:**
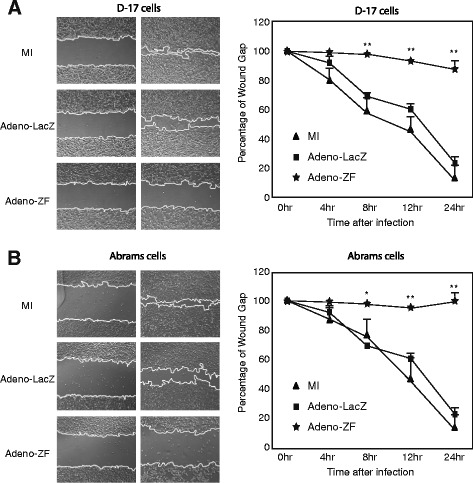
**Ectopic expression of Zhangfei causes decreased ability of canine osteosarcoma cells to repair a scratch wound. (A)** Scratch wounds were made in 100% confluent cultures of D–17 or Abrams cells mock-infected or infected with Adeno-ZF or Adeno-LacZ. Phase contrast images were taken at 0, 4, 8, 12, and 24 hours after infection from identical regions. **(B)** The wound size relative to the starting wound size was measured at each time point after infection in three independent experiments and expressed as a percentage reduction in wound size + standard deviation. Significance of differences of means (*P < 0.05, **P < 0.01) were determined using ANOVA.

Migratory behaviour in cancer cells is a typical hallmark of malignancy. To investigate whether ectopic expression of Zhangfei correlated with altered migratory behaviour, we performed cell motility assays on the canine OS cultures. Following scratch wounding, wound closure was significantly slower in cultures (D–17 and Abrams canine cells) infected with Adeno-ZF compared to cultures infected with Adeno-LacZ or mock-infected cells (Figure 5), showing that the ectopic expression of Zhangfei indeed causes decreased cell motility in canine osteosarcoma cells. However, it is also possible that the decrease in ability of Zhangfei-expressing cells to grow heal the scratch wound may be because of a decrease in growth rates rather than a decrease in the ability to migrate.

### Zhangfei negatively regulates the UPR in canine osteosarcomas

**Figure 6:**
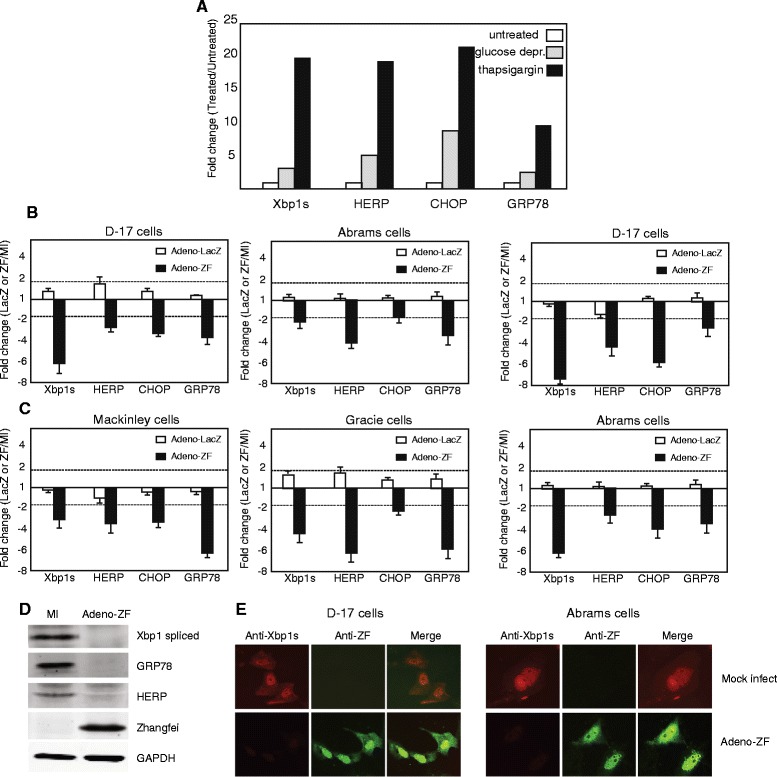
**Zhangfei negatively regulates the Unfolded Protein Responses (UPR) in canine osteosarcomas. (A)** D–17 cells were left untreated, treated with thapsigargin or grown in glucose free medium. Twelve hours later cells were harvested and transcripts for the UPR- linked genes Xbp1s, HERP, CHOP and GRP78 estimated by qRT-PCR. Cells were mock-infected or infected with either Adeno-ZF or Adeno-LacZ and then treated with thapsigargin **(B)** or deprived of glucose **(C)**. 24 hr later cells were harvested and transcripts for UPR genes estimated. Proteins in thapsigargin-treated mock-infected and Adeno-ZF-infected cells were detected by immunoblots **(D)** and immunofluorescence **(E)**. Dotted lines in A and B indicate a 2-fold difference. Values greater than 2 fold were considered significant.

The unfolded protein response (UPR) is an adaptive cellular stress response that alleviates ER stress or, failing, induces apoptosis. In previous studies, we found Zhangfei was a negative regulator of the UPR in D–17 canine OS cells [5]. To investigate if Zhangfei could consistently suppress the UPR in other canine OS cells, the four canine OS cell lines infected with either Adeno-ZF or Adeno-LacZ were treated with the UPR pharmacological inducer thapsigargin, or were deprived of glucose. The latter treatment is a known physiological inducer of the UPR. Both treatments increased the level of transcripts for UPR transcripts Xbp1s, HERP, CHOP and GRP78 (Figure 6A) and Zhangfei suppressed the transcripts in thapsigargin treated (Figure 6B) and glucose deprived (Figure 6C) cells. In contrast, LacZ had no obvious effect. In addition, this decrease in mRNA was reflected in a decrease in UPR proteins (Xbp1s, HERP, and GRP78) in thapsigargin-treated D–17 [5] and Abrams (Figure 6D) cells. Figure 6E, which showed intracellular proteins detected by immunofluorescence, also supported these data—the Xbp1s protein was undetectable in D–17 and Abrams cells expressing Zhangfei.

## Conclusions

Canine OS is an aggressive tumour that accounts for approximately 85% of primary bone tumours in the dog [22]. OS causes local skeletal destruction resulting in osteoproductive and osteolytic lesions, and it is highly metastatic to the lungs. Although there has been dramatic progress in the standard treatments of OS, including amputation, chemotherapy, and palliative radiation therapy, these therapies often fail, leading to recurrence of the tumour and metastatic spread [23-26]. Over the years, combined therapies, such as chemotherapy combined with immune modulators, have been practiced on dog OS [27,28], although with poor overall survival times.

The dog is a well-established model for spontaneous OS in humans, owing to striking similarity in biology and gene expression. The large size of dogs, relative outbreeding, and immunocompetence increase their model potential. Furthermore, dogs with spontaneous tumors naturally develop therapy resistance and metastasis. In addition, tumor burdens in spontaneously arising cancers of dogs are more similar to humans than the experimentally induced tumors found in murine models, which may be important with regard to biologic factors such as hypoxia and clonal variation. The size of canine tumors also allows for serial imaging and tissue collection over time [29].

In previous studies, we found that the transcription factor CREBZF/Zhangfei suppressed the growth of D–17 dog OS cells [5]. Herein, we further discovered that the growth suppressive effects of Zhangfei were applicable to three other independently isolated canine OS cell lines as well.

The unfolded protein response (UPR) is an adaptive response induced by endoplasmic reticulum (ER) stress, which alleviates ER stress by up-regulating the expression of ER-resident chaperons [30], inducing ER-associated protein degradation (ERAD), and down-regulating the synthesis of new proteins [31,32]. If these mechanisms are not sufficient to alleviate ER stress, then an apoptosis program is initiated to induce cell death. Our previous results [15] suggest that Zhangfei is a potential regulator of the UPR, and it might accelerate UPR feedback mechanisms by interacting with the UPR mediator-Xbp1 and targeting it for proteasomal degradation. In the present study, the strong inhibitory effects of Zhangfei on both pharmacological (thapsigargin) and physiological (glucose deprivation) –induced UPR was also observed in the four canine OS cell lines we examined.

Zhangfei suppresses the UPR and cell growth by stabilizing the tumour suppressor protein p53 [6]. All four canine OS cell lines we examined express functional p53 (Figure 1). These results suggest that the induction of Zhangfei expression in canine OS may be an effective strategy for suppressing cell growth and metastasis. However, the strategy would likely only be successful with OS that have functional p53. A large proportion of human cancers have deleted or otherwise non-functional p53. At present we do not know the proportion of canine OS that have inactive p53. Some studies [33] suggest that most canine OS do not have deletions or major rearrangements in the gene for p53. Although 30–50% of p53 coding sequences in canine OS have polymorphisms [33-35] the effect of these changes on p53 functionality is unknown. It is therefore difficult to determine how universally applicable Zhangfei would be as a modality for treating canine OS. The role of p53 in canine OS is also controversial. The expression of ectopic p53 in canine OS cells, both *in vitro* and *in vivo* models leads to reduced tumour growth and an increase in apoptotic cells [5,36,37]. In contrast, other studies in both humans [38] and dogs [39,40] suggest that increased p53 expression in OS correlates with more aggressive tumours and decreases survival time.

## Methods

### Cells and tissue culture

Canine osteosarcoma D–17 cells, obtained from the American Type Tissue Culture Collection, were grown in MEM-Alpha containing 10% fetal bovine serum (FBS). Canine Abrams, McKinley and Gracie cell lines were grown in Dulbecco’s minimal essential medium containing penicillin, streptomycin and 10% newborn calf serum. All media, serum and antibiotics were purchased from Invitrogen (Carlsbad, California). Abrams cells were derived from metastatic OS nodules whereas McKinley and Gracie cells were from primary tumours. All cell lines were confirmed to be of canine origin by multispecies multiplex PCR and identified by short tandem repeat analysis as described [41].

### Adenovirus Vectors Expressing Zhangfei and ß-galactosidase (LacZ)

These vectors were constructed, grown, and purified using the Adeno-X Expression System (Clontech). They were created in our laboratory as described earlier [14]. Cells were infected with Adeno-Zhangfei, Adeno-LacZ (expressing *E. coli* ß-galactosidase, LacZ) or mock-infected. A multiplicity of infection (MOI) of 100 plaque-forming units (pfu) per cell was used.

### WST-1 cell proliferation and viability assay

To determine the growth rate of cells, 10^4^ cells/well were seeded into 96-well plates. 24 h later cells were either mock infected or infected with adenovirus vectors expressing Zhangfei (Adeno-ZF) or ß-galactosidase (Adeno-LacZ). Cell proliferation was assessed using Cell Proliferation Reagent WST-1 (Roche, Mannheim, Germany) according to the manufacturer’s specifications.

### Annexin V-apoptosis assay

Cells were collected after trypsinization and stained with Annexin V and propidium iodide (PI) using an Annexin V kit (Calbiochem) following manufacturer’s instructions. As a positive control cells were treated with 50 μM etopocide (Calbiochem) for 24 hr. Cells were analyzed in a Coulter EPICS XL flow cytometer. In this assay, early apoptotic cells stained with Annexin V but not with PI while late apoptotic or necrotic cells stained with both Annexin V and PI.

### Scratch wound healing assay

Scratch wounds more than 5 mm in length and of equal thickness were made in 100% confluent cultures of D–17 or Abrams cells mock-infected or infected with Adeno-ZF or Adeno-LacZ with a 10 μL disposable micropipette tip. Phase contrast images were obtained at 0, 4, 8, 12, and 24 hours after infection from identical regions. The wound size at each time point after infection relative to the starting wound size was measured using Photoshop software in three independent experiments.

### Quantitative real-time PCR (qPCR)

Total RNA was extracted using RNeasy Plus Mini Kit from Qiagen (Mississauga, ON, Canada). Gene expression was analyzed by RT-PCR using Brilliant II SYBR Green QPCR Master Mix Kit (Agilent Technologies). Levels of GAPDH were used to normalize the samples. All reactions were analyzed in duplicate and each experiment was repeated at least twice. Relative fold changes of transcript levels were calculated as 2^ΔΔ Ct^. Nucleotide sequences of the primers used are in Table 1. The identity of all products was confirmed by electrophoretic mobility on mobility on agarose gels and by determining nucleotide sequence. Only results with homogeneous thermal disassociation profiles were considered.

**Table 1:** **Sequence of primers used for qRT-PCR**

Xbp1	spliced-forward	TCTGCTGAGTCCGCAGCAGG
	spliced-reverse	TAAGGAACTGGGTCCTTCT
HERP	forward	CCGAGCCTGAGCCCGTCACG
	reverse	CTTTGGAAGCAAGTCCTTGA
CHOP	forward	TGGAAGCCTGGTATGAGGAC
	reverse	TGCCACTTTCCTCTCGTTCT
GRP78	forward	GGCTTGGATAAGAGGGAAGG
	reverse	GGTAGAACGGAACAGGTCCA
osteocalcin	forward	AAGCRGGAGGGCAGCAGGT
	reverse	CYGRTARGCYTCCTGRAAGC
GAPDH	forward	TGCCTCCTGCACCACCAACTGC
	reverse	GGGCCATCCACAGTCTTCTGGG

All sequences are in the 5′–3′ direction.

### PCR and sequencing of p53 genes

The sequences of PCR primers used for canine p53 amplification were: canine p53-forward: GGTGACTGCAATGGAGGAGTCGCA, canine p53-reverse: TCAGTCTGAGTCAAGCCCTTCTCT. RNA was purified from cells using the RNEasy Plus mini kit with a genomic DNA elimination step (Qiagen) and RNA converted to cDNA with the Quantitect Reverse Transcription kit (Qiagen) using instructions supplied by the manufacturer. Two-step RT-PCR reactions used TopTaq emzyme (Qiagen) and were performed in a PCR machine. Sequences were aligned using the software, MacVector. Nucleotide sequences for coding sequences for p53 transcripts recovered from D17, Abrams, McKinley and Gracie cell lines were submitted to GenBank/NCBI and were assigned the following accession numbers: KP279761, KP279762, KP279763, KP279764.

### Plasmids and chloramphenicol acetyl transferase (CAT) assay

The construction of pcZF [12], a plasmid that expresses Zhangfei in mammalian cells, has been described. The CAT reporter plasmid pCAT3B-p53RE was constructed by transferring oligonucleotides containing two copies of p53 responsive element, GGTCAAGTTGGGACACGTCCaaGAGCTAAGTCCTGACATGTCT (IDT, Coralville, Iowa), to pCAT3Basic (Promega), which contains the coding sequence for CAT linked to a basal promoter. Oligonucleotides representing the p53 responsive elements with overhanging 5′ terminal KpnI and 3′ terminal BglII sites were annealed and ligated to pCAT3Basic cut with the same enzymes.

In CAT assay, D–17, Abrams, Gracie and McKinley cells were transfected with 0.5 μg of pCAT3B-p53RE, in the presence or absence of a plasmid expressing Zhangfei (pcZF, 1 μg), using Lipofectamine 2000 (Invitrogen) as described in the manufacturer’s instructions. The promoter-less parental reporter plasmid, pCAT3B was included as a control to show basal CAT activity. 250 ng of pCMVBGal, a plasmid specifying β-galactosidase, were added to each transfection as an internal control. 24 h after transfection, the CAT activity was determined by ELISA. CAT values were normalized to β-galactosidase.

### Antibodies, immunoblotting and immunofluorescence

The antibodies used were mouse anti-FLAG (Sigma), rabbit anti-Zhangfei serum [12], rabbit anti-Xbp1 (Abcam, Cambridge, MA), rabbit anti-HERP (Abcam, Cambridge, MA), rabbit anti-GRP78 (Abcam, Cambridge, MA), and mouse anti-GAPDH (Chemicon, Billerica, MA). Suppliers of antibodies against Xbp1, HERP, GRP78 and GAPDH indicated that they recognized canine proteins. Secondary antibodies were goat anti-mouse Alexa488, goat anti-rabbit Alexa546 and goat anti-rabbit Cy5 (Invitrogen). Cells were processed for immunoblotting and immunofluorescence as described previously [5,12].

### Statistical analysis

Statistical analysis of data was performed by Student *T*-test or ANOVA using IBM SPSS statistics version 21.0.0 software. ANOVA tests with LSDpost hoc comparison was used to analyze the differences between multi-group means and their associated procedures by adding individuals as a treatment variable, and a paired *T*-test was used to evaluate the effects of one treatment compared with no treatment/control. A P value of less than 0.05 was considered to be statistically significant for both tests.

#### Availability of supporting data

Accession numbers sequences supporting the results of this article are available in the GenBank repository, [GenBank:KP279761, GenBank:KP279762, GenBank:KP279763, GenBank:KP279764].”

#### Ethics and biosafety statement

All experiments were done following biosafety procedures and precautions approved by the University of Saskatchewan Biosafety committee and under Biosafety Permit VMB-03. All experiment were done on established cell lines and no animals were used.

## Abbreviations

bLZip: Basic leucine zipper
CERBZF: Cyclic AMP response element binding protein Zhangfei
UPR: Unfolded protein response
OS: Osteosarcoma
p53: 53,000 molecular weight protein
Xbp1: X-box binding protein one
ATF4: Activation transcription factor four
SMAD: Homologue of *small body size* (*C. elegans*) and *mothers against decapentaplegic* (*Drosophila*)
VP16: Herpes simplex virus virion protein sixteen
LacZ: Beta galactosidase
GRP78: Glucose responsive protein 78,000 molecular weight
HERP: Homocysteine-responsive endoplasmic reticulum resident protein
CHOP: C/EBP homologous protein
ERAD: Endoplasmic reticulum resident protein degradation
FBS: Foetal bovine serum
MOI: Multiplicity of infection
PI: Propidium iodide
GAPDH: Glyceraldehyde 3-phosphate dehydrogenase
qPCR: Quantitative polymerase chain reaction
CAT: Chloramphenicol acetyl transferase
